# Exercise-Induced Neuroprotection and Recovery of Motor Function in Animal Models of Parkinson's Disease

**DOI:** 10.3389/fneur.2019.01143

**Published:** 2019-11-01

**Authors:** Ewelina Palasz, Wiktor Niewiadomski, Anna Gasiorowska, Adrianna Wysocka, Anna Stepniewska, Grazyna Niewiadomska

**Affiliations:** ^1^Neurobiology Center, Nencki Institute of Experimental Biology, Polish Academy of Science, Warsaw, Poland; ^2^Department of Applied Physiology, Mossakowski Medical Research Centre, Polish Academy of Sciences, Warsaw, Poland

**Keywords:** Parkinson's disease, physical activity, neurotrophic factors, neuroplasticity, dopaminergic system

## Abstract

Parkinson's disease (PD) is manifested by progressive motor, autonomic, and cognitive disturbances. Dopamine (DA) synthesizing neurons in the substantia nigra (SN) degenerate, causing a decline in DA level in the striatum that leads to the characteristic movement disorders. A disease-modifying therapy to arrest PD progression remains unattainable with current pharmacotherapies, most of which cause severe side effects and lose their efficacy with time. For this reason, there is a need to seek new therapies supporting the pharmacological treatment of PD. Motor therapy is recommended for pharmacologically treated PD patients as it alleviates the symptoms. Molecular mechanisms behind the beneficial effects of motor therapy are unknown, nor is it known whether such therapy may be neuroprotective in PD patients. Due to obvious limitations, human studies are unlikely to answer these questions; therefore, the use of animal models of PD seems indispensable. Motor therapy in animal models of PD characterized by the loss of dopaminergic neurons has neuroprotective and neuroregenerative effects, and the completeness of neuronal protection may depend on (i) degree of neuronal loss, (ii) duration and intensity of exercise, and (iii) time elapsed between insult and commencing of training. As the physical activity is neuroprotective for dopaminergic neurons, the question arises what is the mechanism of this protective action. A current hypothesis assumes a central role of neurotrophic factors in the neuroprotection of dopaminergic neurons, even though it is still not clear whether increased DA level in the nigrostriatal axis results from neurogenesis of dopaminergic neurons in the SN, recovery of the phenotype of dopaminergic neurons, increased sprouting of the residual dopaminergic axons in the striatum, or generation of local striatal neurons from inhibitory interneurons. In the present review, we discuss studies describing the influence of physical exercise on the PD-like changes manifested in animal models of the disease and focus our interest on the current state of knowledge on the mechanism of neuroprotection induced by physical activity as a supportive therapy in PD.

## Introduction

Parkinson's disease (PD) is the second most common neurodegenerative disorder. Due to the frequency of occurrence, it is a serious medical, social, and economic problem. Currently, no proven neuroprotective or disease-modifying treatment is available for PD. Several agents can be used to treat the motor symptoms as well as non-motor symptoms, such as depression, fatigue, sleep disorders, and wakefulness, associated with dopamine (DA) deficiency.

Although pharmacological therapy is the current gold standard in the treatment of PD, recent clinical trials have shown that physical activity alleviates and slows down the development of movement impairments, reduces depression and anxiety, and improves mood state, cognitive function, and sleep quality (1, 2). Recent studies have shown that regular physical activity, such as strength training, walking, flexibility, balance, and aerobic training or dance, adjusted to the severity of the disease and to the current PD patient's state of health, is able to enhance brain plasticity, which plays a key role in improving motor and cognitive functions (3).

Physical activity has been found beneficial for persons with PD; however, there is still no answer as to which type, frequency, or intensity of physical exercise is the most effective in relieving Parkinsonian symptoms. The lack of standardized terminology, protocols, interventions, and outcome measures limits the comparison of data and makes them difficult to interpret.

Aerobic training, aimed mainly to increase cardiovascular capacity, has a beneficial effect on PD patients. Reuter et al. (4) study showed that flexibility and relaxation program, walking, and Nordic walking reduced the pain and improved the quality of life of all patients. Nordic walking proved superior in improving postural stability and gait, and other researchers (5–8) confirmed improvement of movement parameters by this kind of walking. On the other hand, Bello et al. (9) demonstrated the superiority of training on the treadmill compared to overground walking in improving gait and body balance. In yet another study, treadmill training was also found to improve speed, cadence, stride length, and distance walked (10).

A common symptom of PD is muscle weakness. The strength (resistance) training increases muscle mass and bone mineral density, sustains body balance, and thus improves the quality of life of PD individuals. Scandalis et al. (11) showed improved gait function in patients with mild to moderate PD subjected to resistance training. Hirsch et al. (12) confirmed a lower fall risk and a longer independent life of PD individuals in response to resistance and balance exercises.

Improvement in motor function can be seen as specific effect of physical training. However, such activity also preserves or improves cognitive function in PD patients, which suggests training to act as a disease-modifying factor. Physical exercise has been shown to improve performance in verbal fluency tests and to reduce spatial working memory errors in cognitive tests. Cruise et al. (13) showed that a combination of strength and cardiovascular training improved executive function in the course of PD. Tabak et al. (14) and Nocera et al. (15) in small case studies observed improvement in executive function in PD patients after aerobic exercises.

**Figure 1:**
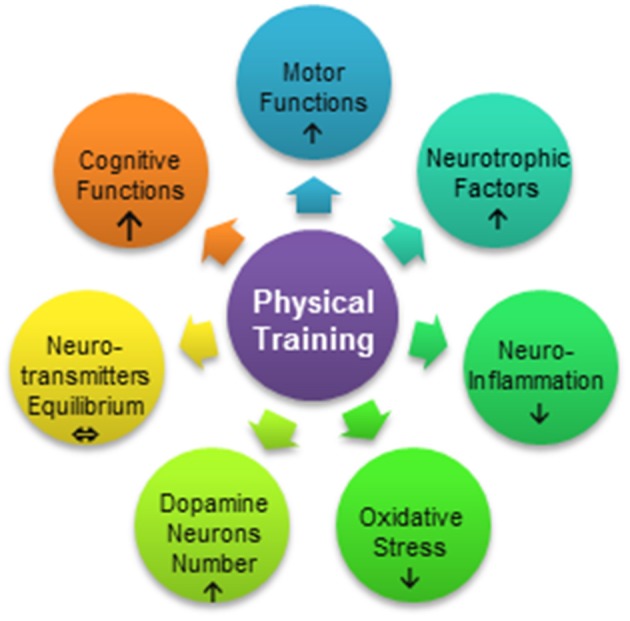
Processes influenced by exercise in Parkinson's disease. Physical training can improve motor function, reduce neuroinflammation, and increase resistance to oxidative stress or reduce the stress level. Motor therapy results in mobilization of neurotrophic factors, protection of dopamine neurons, and restoration of the equilibrium between neurotransmitters such as dopamine and glutamate. Physical exercise may also lead to the enhancement of cognitive functions.

Although the collective evidence supports physical activity as a measure for PD prevention, the mechanism underlying the diminished risk of PD development in physically active persons is still not fully understood (16). Based on animal studies, several mechanisms are believed to explain the effects of physical activity as an adjuvant therapy against PD: increased synthesis and release of neurotrophins (17–20), restoration of the equilibrium between the level and interactions of neurotransmitters (21, 22), increased resistance to oxidative stress (23), reduced inflammatory process in the brain (21, 24, 25), and enhanced synaptogenesis (26), angiogenesis (27), and neurogenesis (28, 29) (Figure 1). In the present review, we aimed to describe the influence of physical exercise on PD symptoms in animal models of the disease and report the current state of knowledge concerning the mechanism underlying the neuroprotective effects of physical activity as supportive therapy in PD.

## Exercise Induces Recovery of Motor Function and Neuroprotection in Animal Models of PD

### Motor Performance

Many studies in animal models of PD showed that various forms of physical activity, differing in type of effort, duration, intensity, and starting point with respect to neurotoxic insult, were neuroprotective and suppressed processes involved in PD pathology (30–32).

The two commonly used toxins leading to the degeneration of nigral dopaminergic neurons and, in consequence, to DA depletion in the striatum are 1-methyl-4-phenyl-1,2,3,6-tetrahydropyridine (MPTP) and 6-hydroxydopamine [6-OHDA]. The most commonly adopted forms of physical activity in animals are the running wheel, performed voluntarily, and forced treadmill training. There are data proving a reduction of motor performance after administration of PD-inducing neurotoxins (30–33) and a protective effect of physical activity (19, 34–36) against behavioral impairments caused by these neurotoxins in animals. However, the detection and experimental quantification of mouse motor impairments that truthfully mimic the symptoms of PD patients has proved difficult. To detect reliably behavioral deficits using standard mouse motor tests such as the rotarod, inverted grid test, and general locomotor activity monitoring in open field, very large doses of MPTP must be used and behavioral alterations often appear to be transient (32, 37, 38). Movement disorders are only slight in mice performing simple motor tasks, even if the loss of dopaminergic neurons in SN after exposure to MPTP ranges from 60 to 70% (39–41), and this may explain why some researchers report no detectable motor deterioration. Nevertheless, it has been shown that physical activity has both protective (42, 43) and restorative (22, 44, 45) effects on motor parameters such as velocity (46), locomotor activity (42), or body balance and coordination (34, 43).

Several studies report that physical exercise led to motor function improvement in rotarod test in a mouse model of PD (47–51). The MPTP-treated groups that completed a treadmill exercise regimen achieved significantly higher maximal velocity than those in the MPTP sedentary group, although there are data showing no effect of MPTP on the rotarod performance (32, 52). A few reports show that mice treated with MPTP have reduced locomotion; however, there are also studies that report no such changes (53–55) as well as reports in which a restorative effect of endurance exercise on locomotion measured in the open field was noticed (42).

Movement in the upside-down position seems to be a task so complex that it should reveal impairments caused by the loss of dopaminergic neurons; therefore, the inverted grid test was introduced by Tillerson et al. (32). During this test, mouse movement on the underside of the horizontal grid is recorded. Tillerson et al. (32, 56) detected sustained behavioral deficits in MPTP-treated mice up to 28 days post-injection, and these deficits were inversely correlated with striatal DA content and expression of the dopamine transporter (DAT), vesicular monoamine transporter 2 (VMAT2), and tyrosine hydroxylase (TH). The inverted grid test was then used by other researchers (57–63), but none of them demonstrated any correlation between motor performance and the degree of dopaminergic neuron loss. Interestingly, in the study that provides an in-depth analysis of many motor parameters (64), no adverse effect of MPTP treatment was noticed.

The detection and experimental quantification of motor impairments in rodents that truthfully mimic the anomalies of Parkinsonian patients proved difficult either due to inadequate behavioral tests or—very likely—because motor impairment takes another form in these animals. So, at present, despite the known ability of physical exercise to promote motoric improvement, our knowledge regarding the most beneficial form, duration, intensity, and frequency of exercise is still insufficient.

### Protection of Dopaminergic Neurons

Depending on when physical training is commenced with respect to neurotoxic insult, one may examine (i) the preventive role of exercise against PD once such insult follows training; (ii) the modification of the course of disease, when the chronic treatment is paralleled with training; and (iii) the neuroregenerative effect of exercise when physical training is applied post-insult.

The neuroprotective role of exercise against the loss of dopaminergic neurons was studied by Gerecke et al. (65), where voluntary physical activity on the running wheel was applied before acute administration of MPTP. The authors determined the critical duration of voluntary exercise necessary for neuroprotection of dopaminergic neurons. They found that 1 month of training provided no neuroprotection, 2 months of training conferred partial protection, and only the first 3 months of training prevented any loss of substantia nigra pars compacta (SNpc) dopaminergic neurons by subsequent neurotoxin treatment. However, beside duration of the training, the distance covered also turned out to be essential. It was found that the longer distance run on the wheel, the lesser loss of DA neuron in the 3-month training group, so only mice that had run the longest distance were completely protected against MPTP toxicity.

The effects of ongoing training on the progression of PD were modeled with daily treadmill running during chronic neurotoxin treatment realized with 10 injections of MPTP spread over 5 weeks. Such chronic MPTP treatment leads to permanent, lasting at least 6 months, neurological deficits resembling, though incompletely, PD. This is unlike acute and subacute MPTP treatments, after which neurological deficits and behavioral changes soon wane spontaneously (65).

Ahmad et al. (66) using this chronic mouse model proved that 10- and 18-week-long treadmill training, started 1 week before commencing MPTP treatment, reduced loss of dopaminergic neurons in the ventral tegmental area (VTA). It should be noted, though, that this model encompasses both parallel application of neurotoxin and physical training as well as post-insult training. Somewhat surprisingly, the number of DA neurons was greater following 18 weeks of exercise training, than 10, which may prove that either new DA neurons appeared and/or some DA neurons that lost their phenotype regained it during subsequent 8 weeks.

Pothakos et al. (42), using the same model, started treadmill training 1 week before MPTP treatment, continued it during intoxication lasting 5 weeks, and over the subsequent 8–12 weeks. They did not observe either the recovery of striatal DA or preservation of TH positive neurons in the SNpc in exercising MPTP-treated mice. It may be important that different doses of MPTP have been used in these studies−12.5 vs. 25 mg/kg of MPTP per injection. The importance of dose was confirmed by Lau et al. (19). In the same chronic model of PD with 18-week treadmill training, which was started 1 week before MPTP intoxication, they observed nearly complete preservation of TH immunopositive neurons in the SNpc and the level of striatal DA comparable to that in control mice. The importance of MPTP dosing was reflected in the loss of 55% of TH positive neurons observed in the study of Lau et al. (19) who used 15 mg MPTP/kg/injection and a 72% loss in the study of Pothakos et al. (42) who applied 25 mg/kg/injection.

Two studies that used the protocol described above delivered contrasting results: Pothakos et al. (42) did not observe either the recovery of striatal DA or preservation of TH positive neurons in the SNpc in exercising MPTP-treated mice, whereas Lau et al. (19) found nearly complete preservation of TH immunopositive neurons in the SNpc and the level of striatal DA comparable to that in control mice. The possible explanation of this discrepancy may be the dose of MPTP, which, in Pothakos et al. study, is twice as big as in Lau et al. study. The difference in MPTP dose was reflected in the 72% loss of nigral DA neurons in the former vs. 55% in the latter study.

Studies on the beneficial effects of exertion applied after neurotoxin application show varying results: from no effect to partial or complete preservation of the number of TH-positive nigral neurons. Aerobic training lasting 4 weeks and started after 5 weeks of treatment with 25 mg MPTP/kg/injection slightly raised nigrostriatal TH and DA levels as compared with sedentary MPTP-injected mice (67). Kintz et al. (22) found that training lasting 37 days, which started 5 days after acute dosing with MPTP, did not return the level of striatal DA reduced by the neurotoxin. Zhao et al. (68) found that the number of nigral dopaminergic neurons returned almost to that observed in control mice after 4 weeks of vibration training following 1 week of MPTP treatment and was also significantly greater than that in sedentary MPTP-treated animals. This was accompanied by similar changes in the striatal DA level. In another study (69), it was found that 8 weeks of progressive treadmill that started 2 weeks after chronic high-dose (25 mg/kg/injection) MPTP administration caused nearly complete restoration of TH-positive neurons in the SNpc, as well as similar recovery of TH and DAT in MPTP-treated exercising mice.

Increased number of dopaminergic neurons observed in neurotoxin-treated animals after physical exercise could be the result of better protection of neurons or neurogenesis. In a study performed by Jang et al. (45), mice were intraperitoneally injected with 25 mg/kg MPTP daily for 1 week. Training on the treadmill lasted 6 weeks and was applied 4 weeks after the last MPTP dose. This training induced neurogenesis in MPTP-treated mice, evidenced by an increased number of bromodeoxyuridine (BrdU)-positive neurons, and attenuated the loss of dopaminergic neurons as manifested by higher levels of TH and DAT. The authors also showed enhanced autophagy, exemplified by changes in autophagy-related protein levels (e.g., microtubule-associated protein 1A/1B-light chain 3—MAP1LC3, nucleoporin p62—p62, Beclin 1, Beclin 2 (Bcl-2)/adenovirus E1B-19 kDa interacting protein—BNIP3, lysosome-associated membrane protein 2—LAMP2, cathepsin L and transcription factor EB—TFEB), and augmented antioxidant capability (e.g., increased level of superoxide dismutase-1—SOD-1, catalase, glutathione peroxidase 1—GPX1, heme oxygenase-1—HO-1, and DJ1) as compared with the results obtained in the MPTP group with no treadmill training.

Also, in a rat model of PD induced by 6-OHDA injections, significant preservation of TH-immunopositive neurons in the SNpc and fibers in the striatum was observed after 4 weeks of treadmill training applied 24 h post-neurotoxin insult (70).

The neuroprotective potency even of a late (i.e., post-neurotoxic insult) physical training was recently illustrated by our study (71), where training of the same duration and intensity attenuated neuronal loss to the same degree, both when commenced before or after chronic MPTP treatment.

Although most reports prove that physical activity positively affects motor functions (19, 34, 44, 47–50, 72) and increases the level of DA in the striatum (19, 73), there are also studies reporting the lack of effects of physical exercise on these parameters (22, 52, 53, 55). For example, Gerecke et al. (65) have found that, even though 3 months of training completely protected dopaminergic neurons against MPTP-induced neurotoxicity, the level of DA and its metabolites in the striatum was significantly lower compared to controls. In order to account for these discrepant findings, the authors hypothesize that uptake of 1-methyl-4-phenylpyridinium (MPP^+^) by DAT generates the formation of hydrogen peroxide and nitrosylated proteins. These compounds damage the dopaminergic terminals but not the cell body. It is also possible that MPP^+^ alone lowers the ability of dopaminergic neurons to transport DA into striatal terminals leading to decreased DA level.

## Regulation of Dopaminergic and Glutamatergic Transmission *via* Physical Activity

Degeneration of dopaminergic neurons in the SNpc and striatal loss of axonal terminals are key pathological features of PD in humans (74–76) and in toxin-induced animal models (19, 42, 71, 77). DA deficiency in PD also leads to loss of dendritic spines within the striatum, which results in motor impairments (78). Two different types of dopaminergic activity can be observed in the striatum: a phasic, brief high-amplitude increase of DA release that acts at the synaptic space through the low affinity D1 receptor (D1R) and a tonic DA release of low amplitude that acts through the high-affinity D2 receptors (D2R) located in the extrasynaptic space. The striatum is functionally subdivided into ventral and dorsal areas, which participate in different aspects of motor control (74). The dorsal striatum is mainly composed of two subpopulations of medium spiny neurons (MSNs): DA D1 receptor-expressing MSNs that constitute the striatonigral or direct pathway (dMSNs) and DA D2 receptor-expressing MSNs that constitute the striatopallidal or indirect pathway (iMSNs). It has been suggested that each pathway has a different role in motor control, with dMSNs being involved in the main aspects of motor control, including motor program selection and activation, and iMSNs in selection and activation of a context-specific motor programs based on the integration of motivational/emotional signaling with sensory-motor inputs (79).

Toy et al. (77) have found a decrease in dendritic spine density in both D1R and D2R containing MSNs after acute MPTP administration and an increase in dendritic spine density and arborization in MSNs of both pathways after 30 days of intensive treadmill exercise. Recovery of dendritic spines was associated with increased expression of post-synaptic density protein 95 (PSD-95) and of presynaptic synaptophysin, leading to increased synaptogenesis in the dorsolateral striatum and improvement in motor performance (77).

The deficiency of DA leads to structural and functional changes in MSNs. These changes usually entail increased glutamatergic projection and hyper-excitability of the indirect pathway (D2R-iMSNs) (80). The prolonged elevated level of glutamate in the intercellular space results in longer depolarization and in disturbances of ionic homeostasis that elicits excitotoxicity and, in consequence, cell death. The α-amino-3-hydroxy-5-methyl-4-isoxazolepropionic-acid receptor (AMPAR), a fast-acting ionotropic glutamate receptor, plays a critical role in these processes. AMPARs are tetrameric channels composed of various combinations of four glutamate receptor subunits, GluA1–GluA4. In particular, the presence or absence of GluA2 decides about important electrophysiological channel properties, including calcium (Ca2+) permeability. Reduced level of GluA2 in AMPAR channels may lead to increased permeability for calcium ions.

Alterations in AMPARs expression have also been noted in animal models of PD characterized by a reduced level of DA. Kintz et al. (22) have noticed that MPTP treatment alone or combined with exercise evoked changes in AMPAR level limited to the D2R-iMSNs striatopallidal pathway. They observed an increase in the level of AMPARs lacking GluA2 in MPTP-treated mice. Increased expression of the GluA2 subunit of the AMPAR in animals that started to exercise after acute MPTP administration suggested the restoration of normal AMPAR subunit expression. Enhanced expression of GluA2-lacking AMPAR possibly potentiates glutamatergic signaling, which leads to hyperexcitability of the striatopallidal projection pathway observed as a consequence of DA depletion (81). Exercise increased the presence of GluA2 subunit in AMPARs in MPTP-treated mice. This could decrease Ca^2+^ influx and diminish glutamatergic drive leading to reduced glutamatergic projection and thus increased survival of dopaminergic neurons.

The hypothesis of increased glutamatergic projection in the course of PD and reduced glutamatergic tonus in response to physical activity has been strengthened by Scone et al. (21), who showed that while levels of vesicular glutamate transporter 1 (VGLUT1) and glutamate transporter-1 (GLT-1) were elevated after MPTP administration, the level of these transporters was decreased following physical activity, restoring glutamate homeostasis in treated mice. Fisher et al. (44) analyzed the neuroprotective effect of 30 days of treadmill training on proportions between dopaminergic and glutamatergic projection in C57 BL/6J mice acutely administered with MPTP. The treadmill training was started 4 days after MPTP or saline treatment. The MPTP exercise group demonstrated significantly reduced DAT immunoreactivity, higher expression of D2R mRNA, and a significant decrease in the density of glutamate terminals compared to the MPTP sedentary group. An increase in the density of nerve terminal glutamate immunolabeling, characteristic for MPTP lesioning, may, therefore, reflect a decrease in the extracellular levels of striatal glutamate. Consequently, the effect of exercise in an MPTP-lesioned brain may be the increased release of glutamate at the synapse, which may alter DA receptor subtype expression or/and function of medium spiny neurons.

## Exercise Mobilizes Neurotrophic Factors

*Post-mortem* examinations of PD brain in human and animal models demonstrate a decreased level of neurotrophic factors (NTFs) in the nigrostriatal pathway (82–84), although this reduction could be partially due to the loss of SNpc dopaminergic neurons, which specifically express one of the NTFs, the brain-derived neurotrophic factor (BDNF). Decreased efficiency of neurotrophic factors, such as BDNF, nerve growth factor (NGF), neurotrophin-3 (NT-3), and neurotrophin-4 (NT-4) in PD may be connected with a reduced level of cyclic nucleotides. Such reduction leads to dysregulation of transcription of NFT encoding genes, mediated by the cyclic adenosine monophosphate (cAMP) response element-binding protein (CREB) (85–87).

NTFs are endogenous proteins that promote differentiation, maintenance, function, and plasticity of the nervous system and allow neurons to survive and repair after injury (88). Therefore, NTFs may serve as potential therapeutic agents in the treatment of neurodegenerative diseases including PD. For example, BDNF, mesencephalic astrocyte-derived neurotrophic factor (MANF), glial cell line-derived neurotrophic factor (GDNF), and cerebral dopamine neurotrophic factor (CDNF) have been shown to be neuroprotective and neurorestorative toward damaged dopaminergic neurons in cell cultures and in various PD animal models (89). *In vivo*, NTFs induce survival of nigrostriatal DA cell bodies and fibers improving motor performance compromised in parkinsonian animals.

The best known trophic factors that protect dopaminergic neurons against oxidative stress are BDNF and GDNF. Initially, the potential therapeutic role of BDNF and GDNF in dopaminergic neurons was found in *in vitro* studies (31, 89, 90). BDNF is widely and abundantly expressed in the brain and significantly involved in several aspects of neuronal development and maturation, plasticity, and recovery mechanisms (91, 92). BDNF also supports the survival of several types of neurons, including mesencephalic dopaminergic, septal cholinergic, and striatal gamma-aminobutyric-acid-releasing (GABAergic) neurons. Binding of BDNF to the high-affinity tropomyosin-related kinase B (TrkB) receptor leads to phosphorylation of TrkB and activation of the three essential downstream intracellular signaling cascades within neuronal somata: phospholipase C-γ (PLCγ), phosphatidylinositol 3-kinase/protein kinase B (PI3K/AKT), and mitogen-activated protein kinase/extracellular signal-related kinase (MAPK/ERK) pathways. Furthermore, BDNF may activate the CREB transcription factor and CREB-binding protein (CBP) and regulate expression of genes encoding proteins involved in stress resistance and cell survival and even neural plasticity (93).

GDNF, in turn, is believed to be the most important neurotrophic factor in the nigrostriatal dopaminergic system and therefore is considered to have therapeutic potential for neuroprotective and regenerative interventions in PD (88). GDNF promotes the survival and maturation of dopaminergic neurons and increases their high-affinity DA uptake (89). It exerts similar effects on motor, adrenergic, parasympathetic, enteric, and somatic sensory neurons (94). GDNF binds with high affinity to glycosylphosphatidylinositol-linked receptor α1 (GFRα1), which is highly expressed in midbrain dopaminergic neurons when measured at mRNA and protein levels. The complex of GDNF and GFRα1, in turn, recruits a transmembrane rearranged during transfection (Ret) receptor and triggers downstream signaling, which controls mitochondrial morphology and complex I activity in dopaminergic neurons (94). GDNF, after binding to its receptor, activates signaling pathways leading to stimulation of dopaminergic neuron excitability, inhibition of DAT activity, and stimulation of TH phosphorylation (95).

There is increasing evidence that NTFs are critical for exercise-induced neuroprotection. The study of Lau et al. (19) has shown that the exercise-induced recovery of cell number and motor behavior in the chronic MPTP mouse model of PD was associated with an improved mitochondrial function and an increase in the brain region-specific levels of BDNF and GDNF. Furthermore, Zhao et al. (68) demonstrated that in MPTP mice, 4 weeks of vibration training almost completely restored dopaminergic neurons in the SN, lost due to MPTP treatment, and DA levels in the striatum, and significantly increased the level of BDNF in the striatum. The authors postulated that long-lasting vibration training could protect dopaminergic neurons from MPTP-induced damage probably by upregulating BDNF. In turn, Gerecke et al. (20), using BDNF^+/−^ mice, showed that exercise was not able to protect dopaminergic neurons from MPTP-induced neurodegeneration.

In another study by Tajiri et al. (70), daily forced treadmill training was used in the 6-OHDA acute rat model of PD. In the 6-OHDA training group, behavioral recovery in the cylinder test and a significant decrease in the number of amphetamine-induced rotations were observed. This was accompanied by the preservation of dopaminergic fibers in the striatum and neurons in the SNpc. In addition, BrdU/doublecortin (Dcx) co-staining revealed enhanced proliferation and migration of neural progenitor cells from the subventricular zone (SVZ) toward the injection site. At the same time, BDNF and GDNF levels were upregulated in the striatum. Physical training in animal models of PD, beside neuroprotection of the dopaminergic system, may enhance neurogenesis and progenitor cell migration through upregulating BDNF-TrkB and GDNF-Ret signaling, which can, in turn, stimulate certain signaling cascades, including those that activate the PI3K/Akt pathway or the extracellular signal-regulated kinases 1 and 2 (ERK1/2) cascade. The PI3K/Akt signaling pathway transmits anti-apoptotic signals stemming from neurotrophins. This pathway activates transcription factors such as CREB and nuclear factor kappa-light-chain-enhancer of activated B cells (NF-kB), which trigger the expression of genes involved in cell survival, such as Bcl-2 and other inhibitors of apoptosis. It also suppresses the expression of pro-apoptotic genes, such as the Bcl-2-associated death promoter (BAD) and Forkhead box (FOX) transcription factors (96). Similarly, the ERK1/2 signaling cascade induces anti-apoptotic genes such as those encoding Bcl-2 and the transcription factor CREB. ERK1/2 also mediates neuritic outgrowth induced by neurotrophic factors (97) and phosphorylation of TH, which increases its enzymatic activity, thus enhancing DA synthesis (98).

Additionally, physical activity, through the induction of neurotrophin signaling pathways, affects synaptic plasticity in MPTP mice. In the study of Zhu et al. (99), the authors have demonstrated that long-term potentiation (LTP) impairment in the MPTP model was due to the decrease in hippocampal BDNF level. Both induction of endogenous BDNF synthesis by memantine or application of exogenous BDNF restored the LTP deficit in the MPTP model. In addition to the effects of memantine on LTP, MPTP-enhanced long-term depression (LTD) was also reversed by memantine administration. These data suggest that compounds that can activate BDNF synthesis have the potential to protect memory in PD.

The evidence that exercise, through regulation of growth factors, secures successful brain function was demonstrated in several other studies (100–104). Also in our study (71), the results suggest that 10 weeks of training on the treadmill, no matter if started before or after PD induction, have a protective effect against dopaminergic neuron degeneration in the chronic MPTP mouse model of PD. Neuroprotective properties of exercise were most likely associated with increased expression of endogenous NTFs and reduced inflammation in the brain. Although the actual sequence of events needs to be discerned, it is likely that NTFs protect neurons against degeneration and in this way prevent the inflammatory response. However, the other way is also possible, i.e., that the strength of inflammation is regulated by the neurotrophin signaling pathways irrespective of neurodegeneration. It is likely that both ways operate when training accompanies neurotoxic assault and also when it is applied after such assault. In summary, these studies underscore the view that physical activity remains neuroprotective even during the advanced stage of PD and therefore provide strong support for starting and continuing physical activity at any point of the disease.

## Physical Training Modulates Neuroinflammatory Mechanisms

Although the death of dopaminergic neurons is a well-characterized pathological feature of PD, the primary cause of the disease is still not clear. Since the first observations of reactive microglia and astrocytes in *post-mortem* brain samples of PD patients were made (105, 106), numerous studies have proposed a role of glial cells in the neuropathology of PD (107–109). Furthermore, infiltration of cells of the peripheral immune system into the brain is also likely to play a role in the pathomechanism of PD (110, 111). It is not clear whether inflammation is a primary cause of PD or a secondary event, that is, the consequence of neuronal death. Short-lasting inflammatory reactions may induce NTFs (112, 113) and may protect neurons against reactive oxygen species (ROS) (114) and glutamate neuronal hyperexcitability (115) but chronic inflammation usually leads to degeneration and neuronal damage (113, 116, 117).

Neuroinflammation means activation of microglia and reactive astrocytes, which, in turn, produce cytokines, chemokines, reactive oxygen, and nitrogen species, secondary messengers, prostaglandins, and protein complement cascades (118). Elevated levels of interleukin-6 (IL-6) acting as pro-inflammatory cytokine, tumor necrosis factor alpha (TNF-α), interleukin-1 beta (IL-1β), and nitric oxide synthase (NOS), found in the SN, putamen, as well as in the cerebrospinal fluid (CSF) and serum of PD patients suggest that, in PD, glial cells acquire a pro-inflammatory phenotype (119).

Both the pro-inflammatory cytokines and their receptors play a role in the pathogenesis of PD. A glial reaction in the brain involving astrocytes and microglia have been described in several toxin-induced animal models of PD (21, 46, 120, 121). Loss of dopaminergic neurons is associated with activated microglia (122–124), which, in turn, activates astrocytes. Increasing evidence suggests that the CD200 protein and its receptor, CD200R, play a critical role during microglia activation. Deficits in the CD200–CD200R pathway exacerbate microglial activation and degeneration of dopaminergic neurons (125).

Liddelow et al. (126) have shown in *in vitro* and *in vivo* studies that activated microglia stimulate, in turn, astrocytes by secreting interleukin-1 alpha (IL-1α), TNFα, and complement component 1q (C1q). Lofrumento et al. (109) have observed a significant increase in IL-1β, TNF-α, and IL-6 mRNA expression level and an increase in both mRNA and protein levels of their respective receptors IL-1RI (IL-1 receptor type I), TNF-αRI, and IL-6Rα in the SN of MPTP-treated animals in comparison with untreated mice.

Astrogliosis is characterized mainly by an increase in the number and size of astrocytes and increased expression of the glial fibrillary acidic protein (GFAP). Due to pro-inflammatory activation, astrocytes lose the ability to promote neuronal survival, neurite outgrowth, synaptogenesis, and phagocytosis (127). Astrogliosis has been confirmed during PD (35, 128). Sconce et al. (21) have shown an increase in GFAP immunoreactivity and a reduced ratio of phosphorylated to non-phosphorylated component 3 of the nuclear factor of activated T-cells (NFATc3) within the SN in MPTP mice.

The neuroprotective effect of physical activity has been linked to the prevention and modulation of the inflammatory process (129). Recent evidence suggests that physical activity, in addition to modifying the cardiovascular, muscular, and hormonal systems, also affects the immune system, which can lead to the overall anti-inflammatory effect in the whole body (130), including attenuation of the inflammatory processes in the brain in the course of neurodegenerative diseases.

Sconce et al. (21) have reported that running wheel exercise affects behavioral deficits, as well as dopaminergic, glutamatergic, and inflammatory biomarkers in a progressive MPTP mouse model of PD. The authors noticed recovery of motor abilities, increased VMAT2 expression, decreased glycosylated-DAT expression, reduced levels of VGLUT1 and GLT-1, and lower levels of the inflammatory marker, component 3 of the nuclear factor of activated T-cells (NFATc3), and of the astrocytic marker, GFAP, in MPTP/exercised mice as compared to MPTP mice without exercise. The anti-inflammatory effect of different types of physical activity has been verified by Goes et al. (24). The authors found that 4-week swimming training alleviated cognitive and motor impairment, and prevented both the increase of ROS and IL-1β levels and inhibition of glutathione peroxidase (GPx) glutathione *S*-transferase (GST) and glutathione reductase (GR) activities. This training also restored DA, 3,4-dihydroxyphenylacetic acid (DOPAC), and homovanillic acid (HVA) activity levels in the striatum of mice administered with 6-OHDA (24).

Recent studies have suggested that interaction between alpha-synuclein (α-syn) and toll-like receptor 2 (TLR2) may play a critical role in the onset of neuroinflammation (131). Jang et al. (132) observed in mice that 8-week treadmill training started after completion of chronic MPTP treatment eliminated motor coordination deficits, increased nigrostriatal TH level, and diminished expression of α-syn in the striatum. This, in consequence, led to downregulation of TLR2 signaling molecules such as myeloid differentiation primary response 88 (MyD88), tumor necrosis factor receptor-associated factor 6 (TRAF6), and phosphorylated transforming growth factor-β-activated protein kinase 1 (p-TAK1). What was interesting, physical activity significantly decreased GFAP level and increased the level of phosphorylated NFATc3, thus reducing the susceptibility of dopaminergic neurons to apoptosis.

In another study (71), the immunohistochemical staining and ELISA assay against GFAP showed an increased number of astrocytes and elevated GFAP concentration in SNpc and VTA in MPTP sedentary mice. On the other hand, treadmill training caused reduced GFAP concentration in the MPTP-trained group, comparable to the concentration of GFAP observed in the control groups. Furthermore, staining against CD11b and ionized calcium-binding adapter molecule 1 (Iba1), the markers of microglia, also showed higher intensity in the SNpc and VTA of MPTP mice without treadmill training compared with results obtained in controls and the MPTP group with treadmill training (71).

Both subtypes of glial cells, astrocytes and microglia, may be activated in two different ways, resulting in a pro-inflammatory (classical M1 activation) or anti-inflammatory (alternative M2 activation) formation of response. In the latter case, stimulated microglia show increased expression of cytokines recognized as an anti-inflammatory, such as interleukin-10 (IL-10), transforming growth factor beta (TGFβ), insulin growth factor 1 (IGF-1), NGF, and BDNF (133). Astrocytes, similarly to microglia, secrete anti-inflammatory compounds, among them neurotrophic factors such as GDNF, BDNF, and MANF, which revive injured dopaminergic neurons and uphold their survival (134, 135).

It is likely that physical training applied in MPTP mice not only reduces M1-type pro-inflammatory glial activation but also promotes M2-type neuroprotective activation of microglia. The increased synthesis of trophic factors observed after prolonged physical training could favor the neuroprotective pathway of microglia activation. In consequence, dopaminergic neurons protected by neurotrophins do not degenerate and thus do not send signals that mobilize the pro-inflammatory response and proliferation of glial cells. Training may reduce the reactivity of microglia and mitigate inflammation by altering multiple metabolic and transcriptional processes (136). For instance, it may inhibit the activity of glycogen synthase kinase 3 (GSK-3), which is a major regulator of the balance between the pro- and anti-inflammatory mediators in immune cells, including microglia (137). GSK-3 stimulates activated microglia to release pro-inflammatory cytokines such as IL-1β, IL-6, and TNFα, and inhibits the release of anti-inflammatory cytokines such as IL-10 (138–140). The way the exercise affects GSK-3 activity may rely on the activation of some extracellular factors, including those mediated by BDNF (141, 142) and GDNF (143, 144), which are known to inhibit GSK-3.

## Physical Training Attenuates Mitochondrial Dysfunction and Oxidative Stress

It has long been recognized that oxidative stress may be important in the etiology of a variety of late-onset neurodegenerative diseases including PD (145–147). Oxidative stress is connected with the production of ROS, which show strong oxidizing properties. Although ROS at physiological concentrations play a pivotal role in several cellular signaling pathways such as cell cycle regulation, phagocytosis, and enzyme activation, excessive generation of ROS leads to several harmful effects including damage to DNA, lipids, and proteins (148).

In the PD brain, the nigral polyunsaturated free fatty acid level is reduced, while the nigral level of lipid peroxidation markers (malondialdehyde and 4-hydroxynonenal) is elevated (149). Also, protein oxidative damage is evidenced by the presence of protein carbonyls (150). Some results suggest that reactive nitrogen species contribute to PD etiology due to their role played in nitration and nitrosylation of certain proteins (151). Oxidative stress leading to cell death may occur in the SNpc because of (i) augmented production of H_2_O_2_ due to increased DA turnover, (ii) reduced brain capacity to eliminate H_2_O_2_ because of glutathione (GSH) deficiency, or (iii) promotion of ^•^OH formation caused by elevated level of the reactive iron (152, 153). Synthesis of neuronal GSH requires delivery of GSH precursors, which mainly arise from extracellular cleavage of astrocyte-derived GSH. Lower nigral levels of GSH have been found in *post-mortem* PD patient brains when compared with age-matched controls. Diminished GSH release from astrocytes may be caused by depletion of reduced GSH in astrocytes, which in turn is caused by increased oxidant production by nicotinamide adenine dinucleotide phosphate (NADPH) oxidase (NOX) (154).

Mitochondria are a key point for programmed cell death (PCD) where apoptosis may be triggered by several factors produced as a result of intracellular stress, such as ROS and calcium ions (Ca^2+^) overload (155). It has been reported that, due to oxidative stress, there are more deletions in mitochondrial DNA of the surviving nigral dopaminergic neurons of PD patients (156, 157). The hypothesis of mitochondrial dysfunction in PD was strengthened after the discovery of mutations in several genes encoding mitochondrial proteins that give rise to a familial form of PD (e.g., *PINK1* and *PARK2, DJ-1*) (158).

The involvement of mitochondrial dysfunction and mitochondrial ROS production has been demonstrated also in experimental animal models using either toxin (MPTP) or genetically altered mice. The *post-mortem* examination of SNpc in PD patients showed a significant increase in the H-subunit and a significant reduction in the L-subunit of ferritin, which was associated with increased iron concentration, ROS overproduction, and neuronal death (159). A reduction in complex I activity has been demonstrated in the SN, lymphocytes, and platelets of PD patients (160). Moreover, increased oxidative stress has been detected in both the rotenone and MPTP-induced toxin models and in genetic models of PD (160, 161). Studies in animals have indicated that MPP^+^, the active metabolite of MPTP, and rotenone inhibit ATP synthesis by blocking electron transport. This blockade is due to binding to complex I. MPP^+^, similarly to rotenone, produces superoxide anions in electron transport particles, which supports the view that MPP^+^ is primarily a mitochondrial toxin (162). The neurotoxic effects of MPP^+^ can be effectively prevented by antioxidants, indicating that the neurotoxicity of this compound is due to oxidative stress (163).

Many studies demonstrate a positive effect of physical exercise on inhibition of oxidative stress (47, 69, 164–172). Using a chronic mouse model of PD, Patki, and Lau (173) revealed that 18-week treadmill training inhibited cytochrome c release, elevated levels of p53, and upregulated the expression of mitochondrial transcription factor A (THAM) and of peroxisome proliferator-activated receptor gamma coactivator 1α (PGC-1α), which are known to be associated with mitochondrial dysfunction and loss of dopaminergic neurons.

Koo et al. (69, 174) demonstrated, in a MPTP/probenecid-induced mouse model of PD, that treadmill training improved mitochondrial function and promoted autophagy *via* the sirtuin-1 (SIRT1) signaling pathway causing α-syn level to decrease. This subsequently attenuated the loss of dopaminergic neurons due to reducing apoptotic cell death mediated by α-syn. Most importantly, training increased expression of SIRT1 that, in turn, enhanced mitochondrial biogenesis and reduced oxidative stress by activating PGC-1α. Furthermore, activation of SIRT1-activated microtubule-associated protein 1 light chain 3 (MAP1LC3) and, in consequence, promoted autophagic clearance of α-syn. Improved mitochondrial function and augmented autophagy caused by physical training were accompanied by ameliorated motor abilities in mice chronically treated with MPTP. Koo et al. (174) have also demonstrated that training on the treadmill, applied after MPTP administration and lasting 8 weeks, reduced the loss of dopaminergic neurons, altered the level of proteins involved in apoptosis (increased Bcl-2 and decreased Bcl-2-associated X protein-Bax and caspase-3), increased expression of core proteins of the translocase of the outer mitochondrial membrane (TOM) complex, which is part of the mitochondrial import machinery (MIM), and increased the level of mitochondrial electron transport chain proteins (cyclooxygenase—COX-I, COX-IV, and mitochondrial 70 kDa heat shock protein—mtHSP70).

In the Al-Jarrah et al. (175) study, the authors have shown a positive impact of exercise training on the concentration of nitric oxide in the striatum of a chronic MPTP mouse model of PD. The 4-week training on the treadmill started after MPTP injections significantly decreased the level of neuronal nitric oxide synthase (nNOS) and inducible form of NOS (iNOS) in animals subjected to MPTP administration and treadmill training compared with the MPTP sedentary group. Four weeks of treadmill training improved the motor skills, evaluated through analyzing gait on the CatWalk, in a unilateral 6-OHDA rat model of PD (167, 168). This training also improved dopaminergic neuron viability, recovered mitochondrial function, and attenuated oxidative stress in PD rats. It was suggested that this phenomenon may be associated with improved mitochondrial turnover, that is, mitochondrial fusion, fission, and clearance, giving rise to increased quantities of mitochondria. It was demonstrated that exercise prevented 6-OHDA-induced loss of TH immunolabeling and activated the nuclear factor (erythroid-derived 2)-like 2 (Nrf2)–antioxidant response element (ARE) axis (i.e., ARE-dependent transcription) in the nigrostriatal pathway in C57BL/6 mice (176). The Nrf2-ARE is a major cellular defense mechanism against oxidative stress, which regulates the expression of antioxidant enzymes, such as gamma-glutamylcysteine ligase (γGCL) and HO-1. The Nrf2-ARE signaling pathway is strongly involved in neuroprotection and anti-inflammatory response. Treadmill training also stimulated mitochondrial biogenesis in the striatum of animals that were more resistant to the oxidant, 6-OHDA, and a nitric oxide donor, namely, (±)-*S*-nitroso-*N*-acetylpenicillamine. Similar results were obtained in the study of Tsou et al. (172) who showed that exercise enhanced the nigrostriatal Nrf2-mediated antioxidant defense capacity to protect dopaminergic neurons against MPP^+^-induced toxicity in mice.

The study, designed to investigate the potential neuroprotective effect of swimming training in a mouse model of PD induced by 6-OHDA (24), demonstrated that a 4-week-long training attenuated the following features associated with PD: increased number of falls in the rotarod test, impairment of long-term memory in the object recognition test, depressive-like behavior in the tail suspension test, and, at the same time, increase in ROS and IL-1β levels, inhibition of GPx activity, increase in GR and GST activities, and decrease in DA, HVA, and DOPAC levels.

Altogether, the findings presented in the above sections indicate that the beneficial effects induced by exercise, when neurodegenerative diseases, such as PD, are concerned, are mostly due to attenuation of oxidative stress and inflammation and elevation of NTFs.

## Conclusions

Epidemiological data support the notion of beneficial effects of physical activity on preventing the risk, development, and progression of PD. Due to the fact that the effectiveness of pharmacological treatment decreases with the development of the disease and because properly selected physical training does not cause side effects, the physical activity should be recommended, as a supportive therapy, to patients suffering from PD. The physical activity not only reduces motor impairments but also improves cognitive functions.

**Figure 2:**
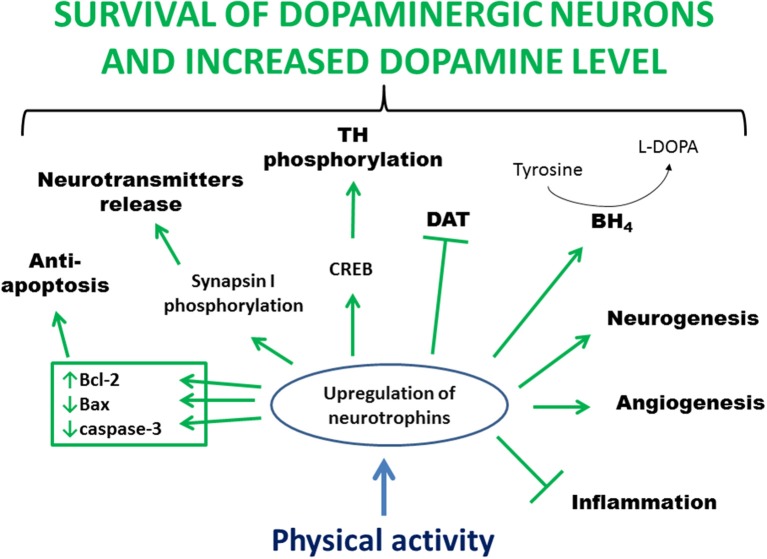
Main processes involved in exercise-induced and neurotrophin-mediated increased viability of dopaminergic neurons. Physical activity leads to an increased level of neurotrophins in the brain. This upregulation stimulates anti-apoptotic proteins, mediates clustering and release of synaptic vesicles, activates CREB leading to phosphorylation and activation of TH, increases BH_4_ level allowing conversion of tyrosine to L-DOPA, activates a pathway that inhibits DAT activity, increases blood flow and angiogenesis, enhances neurogenesis, and reduces inflammation. In summary, all these processes lead to greater survival of dopaminergic neurons and increased level of dopamine. Bcl-2, B-cell lymphoma 2; BH_4_, tetrahydrobiopterin; CREB, cAMP response-element binding protein; DAT, dopamine transporter; L-DOPA, L-3,4-dihydroxyphenylalanine; TH, tyrosine hydroxylase.

Studies in animal models of PD indicate that physical activity may prevent the loss of, protect, or restore dopaminergic neurons probably by activating signaling cascades triggered by the increased availability of neurotrophins (Figure 2). Furthermore, neurotrophins can stabilize intracellular calcium concentration, induce antioxidant enzyme expression, and suppress the release of proinflammatory cytokines. Neurotrophic factors also provide important extracellular signals regulating neurogenesis in the adult brain. Finally, exercise improves motor circuitry through alterations in DA and glutamate neurotransmission.

However, animal models utilizing neurotoxins do not completely reproduce clinical symptoms and pathologies of PD in humans. Therefore, when MPTP is administered to animals, we are talking about inducing parkinsonism, not systemic PD. MPTP animal models are most often used to study the pathogenesis and progression of the disease and the effectiveness of drugs. Among the disadvantages of the animal model of PD caused by MPTP administration, the short-term and acute nature of pathological changes is mentioned. This disadvantage is remedied by the method of administration of neurotoxin used in chronic models of induction of parkinsonism in which the loss of dopaminergic neurons persists for at least several months. Another disadvantage is the difficulty or even impossibility to reproduce the characteristic movement disorders seen in humans. Lack of these disorders can be caused by a different organization of movement control by the nervous system in human and in mice. In animals, motor control is not so highly hierarchical and there is lesser contribution of the cerebral cortex and subcortical structures to locomotion than in humans.

It is likely that neurotoxin-based animal models of PD are adequate experimental models to study aspects of the beneficial effects of physical exertion, concerning changes at the tissue and cellular level and may be well**-**used to investigate such phenomena as neuroprotection, induction of trophic factors and neurogenesis, alleviation of inflammation, reduction of oxidative stress, and improvement of mitochondrial function. On the other hand, these models seems not so useful in studying the effect of exercise on alleviating movement disorders, because the results of the latest studies indicate that the loss of motor function after administration of neurotoxins is insignificant and difficult to capture/notice in the behavioral tests used.

It is possible that the outline, as presented in this review, of mechanisms by which physical activity confers neuroprotection is valid. However, more research is needed to confirm these mechanisms and elucidate their details. There is also a question what is the optimal type, intensity, and duration of the exercise when it should be performed to be maximally effective.

Specifically, as the majority of data supporting the ability of physical activity to protect DA neurons stems from neurotoxin PD models, the question arises whether this property of physical activity would persist in other animal models of PD. With a positive answer to this question, the next issue to be cleared would be whether there are common neuroprotective mechanisms. The existence of such common mechanisms will be strongly supportive to continue physical activity by PD patients during the whole course of illness as a disease-modifying factor.

## Author Contributions

EP, WN, and GN contributed equally to this work and wrote the main body of the paper. AG wrote the Introduction section. AW and AS made the figures and create the references. All authors reviewed the final manuscript.

### Conflict of Interest

The authors declare that the research was conducted in the absence of any commercial or financial relationships that could be construed as a potential conflict of interest.
